# 1,6-Bis(diphenyl­arsino)hexa­ne

**DOI:** 10.1107/S1600536809036757

**Published:** 2009-09-30

**Authors:** Omar bin Shawkataly, Imthyaz Ahmed Khan, Jia Hao Goh, Hoong-Kun Fun

**Affiliations:** a Chemical Sciences Programme, Centre for Distance Education, Universiti Sains Malaysia, 11800 USM, Penang, Malaysia; b X-ray Crystallography Unit, School of Physics, Universiti Sains Malaysia, 11800 USM, Penang, Malaysia

## Abstract

The title diphenyl­arsino compound, C_30_H_32_As_2_ or Ph_2_As(CH_2_)_6_AsPh_2_, lies about a crystallographic inversion centre located at the mid-point of the central C*sp* ^3^—C*sp* ^3^ bond of the methyl­ene chain. The two benzene rings bonded to As are inclined to one another at a dihedral angle of 75.98 (8)°. In the crystal structure, weak inter­molecular C—H⋯π inter­actions stack the mol­ecules down the *b* axis.

## Related literature

For general background to and applications of diphenyl­arsino derivatives, see: Hill *et al.* (1983 ▶); Song *et al.* (2005 ▶). For the preparation of the title compound, see: Aguiar & Archibald (1967 ▶); Burfield *et al.* (1977 ▶, 1978 ▶); Tzschach & Lange (1962 ▶). For closely related structures, see: Hill *et al.* (2001 ▶); Shawkataly *et al.* (2005 ▶). For information on the Cambridge Structural Database, see: Allen (2002 ▶). For bond-length data, see: Allen *et al.* (1987 ▶). For the stability of the temperature controller used for the data collection, see: Cosier & Glazer (1986 ▶).
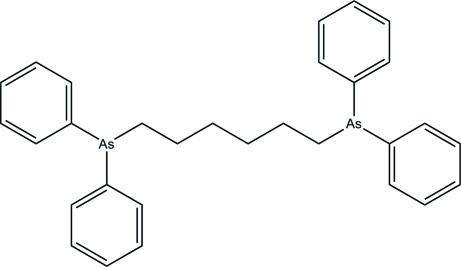


## Experimental

### 

#### Crystal data

C_30_H_32_As_2_
*M* *_r_* = 542.40
Monoclinic, 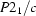

*a* = 12.3774 (2) Å
*b* = 5.7145 (1) Å
*c* = 18.1263 (3) Å
β = 101.076 (1)°
*V* = 1258.20 (4) Å^3^
*Z* = 2
Mo *K*α radiation
μ = 2.67 mm^−1^
*T* = 100 K
0.44 × 0.29 × 0.03 mm

#### Data collection

Bruker SMART APEXII CCD area-detector diffractometer
Absorption correction: multi-scan (**SADABS**; Bruker, 2005 ▶) *T*_min_ = 0.384, *T*_max_ = 0.919
24043 measured reflections
5572 independent reflections
4144 reflections with *I* > 2σ(*I*)
*R*_int_ = 0.052

#### Refinement

*R*[*F* ^2^ > 2σ(*F* ^2^)] = 0.035
*wR*(*F* ^2^) = 0.084
*S* = 1.03
5572 reflections
209 parameters
All H-atom parameters refined
Δρ_max_ = 0.58 e Å^−3^
Δρ_min_ = −0.47 e Å^−3^

### 

Data collection: *APEX2* (Bruker, 2005 ▶); cell refinement: *SAINT* (Bruker, 2005 ▶); data reduction: *SAINT*; program(s) used to solve structure: *SHELXTL* (Sheldrick, 2008 ▶); program(s) used to refine structure: *SHELXTL* molecular graphics: *SHELXTL*; software used to prepare material for publication: *SHELXTL* and *PLATON* (Spek, 2009 ▶).

## Supplementary Material

Crystal structure: contains datablocks global, I. DOI: 10.1107/S1600536809036757/sj2648sup1.cif

Structure factors: contains datablocks I. DOI: 10.1107/S1600536809036757/sj2648Isup2.hkl

Additional supplementary materials:  crystallographic information; 3D view; checkCIF report

## Figures and Tables

**Table 1:** Hydrogen-bond geometry (Å, °)

*D*—H⋯*A*	*D*—H	H⋯*A*	*D*⋯*A*	*D*—H⋯*A*
C15—H15*B*⋯*Cg*1^i^	0.97 (3)	2.81 (3)	3.776 (2)	169.9 (18)
C4—H4⋯*Cg*2^ii^	0.91 (2)	2.80 (2)	3.708 (2)	173.2 (19)
C9—H9⋯*Cg*2^iii^	0.91 (2)	2.97 (2)	3.617 (2)	129.5 (16)

Symmetry codes: (i) 
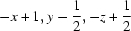
; (ii) 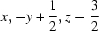; (iii) 
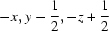
. *Cg*1 and *Cg*2 are the centroids of the C1–C6 and C7–C12 benzene rings, respectively.

**Fig. 1.**
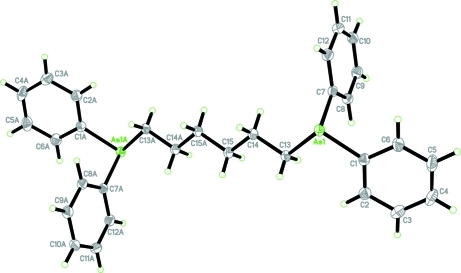
The molecular structure of the title compound, showing 50% probability displacement ellipsoids for non-H atoms and the atom-numbering scheme. The suffix A corresponds to the symmetry code [-x+1, -y+1, -z+1].

**Fig. 2.**
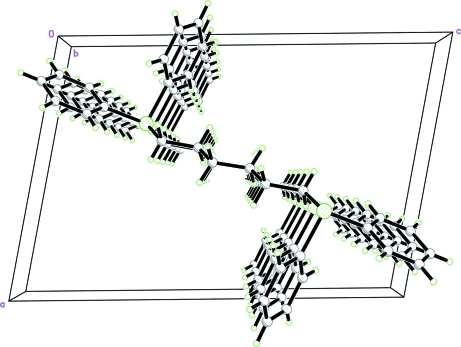
The crystal structure of the title compound viewed along the b axis, showing stacking of molecules along the b axis.

## supplementary crystallographic information

### Comment

1,6-Bis(diphenylarsino)hexane has been used for trans chelation in transition metal complexes (Hill *et al.*, 1983). A search of the November 2008 release of the Cambridge Structural Database (Allen, 2002) revealed no structures of complexes containing the above ligand. Among bis(diphenylarsino)alkanes, only the structure of 1,2-bis(diphenylarsino)ethane (Hill *et al.*, 2001) and complexes of the ligand 1,3-bis(diphenylarsino)propane (Song *et al.*, 2005) are known.

The title compound (Fig. 1), contains a crystallographic inversion centre at the mid-point of the central C*sp*^3^—C*sp*^3^ (C15—C15A) bond [symmetry code of atoms labellel with suffix A: -x+1, -y+1, -z+1]. The C1-C6 and C7-C12 benzene rings are inclined to one another, with a dihedral angle of 75.98 (8)°. The bond lengths (Allen *et al.*, 1987) are comparable to those found in closely related structures (Hill *et al.*, 2001; Shawkataly *et al.*, 2005). In the crystal structure (Fig. 2), the molecules are stacked down the *b* axis. The crystal structure is consolidated by intermolecular C15—H15B···*Cg*1, C4—H4···*Cg*2 and C9—H9···*Cg*2 interactions (Table 1).

### Experimental

Solvents were dried by recommended literature routes (Burfield *et al.*, 1977, 1978) and the title compound was prepared by the reaction of diphenylarsino lithium with 1,6-dibromohexane in dry THF at 273 K under nitrogen atmosphere (Aguiar *et al.*, 1967; Tzschach & Lange, 1962). Colourless plates of suitable quality for single crystal X-ray diffraction studies were obtained by slow evaporation from ethanol solution.

### Refinement

All the H atoms were located from difference Fourier map and allowed to refine freely [range of C—H = 0.89 (2) – 0.99 (3) Å].

### Crystal data

**Table:**

C_30_H_32_As_2_	*F*(000) = 556
*M**_r_* = 542.40	*D*_x_ = 1.432 Mg m^−^^3^
Monoclinic, *P*2_1_/*c*	Mo *K*α radiation, λ = 0.71073 Å
Hall symbol: -P 2ybc	Cell parameters from 5477 reflections
*a* = 12.3774 (2) Å	θ = 3.1–32.2°
*b* = 5.7145 (1) Å	µ = 2.67 mm^−^^1^
*c* = 18.1263 (3) Å	*T* = 100 K
β = 101.076 (1)°	Plate, colourless
*V* = 1258.20 (4) Å^3^	0.44 × 0.29 × 0.03 mm
*Z* = 2	

### Data collection

**Table:**

Bruker SMART APEXII CCD area-detector diffractometer	5572 independent reflections
Radiation source: fine-focus sealed tube	4144 reflections with *I* > 2σ(*I*)
graphite	*R*_int_ = 0.052
φ and ω scans	θ_max_ = 35.1°, θ_min_ = 2.3°
Absorption correction: multi-scan (*SADABS*; Bruker, 2005)	*h* = −19→20
*T*_min_ = 0.384, *T*_max_ = 0.919	*k* = −9→8
24043 measured reflections	*l* = −29→26

### Refinement

**Table:**

Refinement on *F*^2^	Primary atom site location: structure-invariant direct methods
Least-squares matrix: full	Secondary atom site location: difference Fourier map
*R*[*F*^2^ > 2σ(*F*^2^)] = 0.035	Hydrogen site location: inferred from neighbouring sites
*wR*(*F*^2^) = 0.084	All H-atom parameters refined
*S* = 1.03	*w* = 1/[σ^2^(*F*_o_^2^) + (0.0344*P*)^2^ + 0.3375*P*] where *P* = (*F*_o_^2^ + 2*F*_c_^2^)/3
5572 reflections	(Δ/σ)_max_ = 0.001
209 parameters	Δρ_max_ = 0.58 e Å^−^^3^
0 restraints	Δρ_min_ = −0.47 e Å^−^^3^

### Special details

**Table:**

Experimental. The crystal was placed in the cold stream of an Oxford Cyrosystems Cobra open-flow nitrogen cryostat (Cosier & Glazer, 1986) operating at 100.0 (1)K.
Geometry. All esds (except the esd in the dihedral angle between two l.s. planes) are estimated using the full covariance matrix. The cell esds are taken into account individually in the estimation of esds in distances, angles and torsion angles; correlations between esds in cell parameters are only used when they are defined by crystal symmetry. An approximate (isotropic) treatment of cell esds is used for estimating esds involving l.s. planes.
Refinement. Refinement of F^2^ against ALL reflections. The weighted R-factor wR and goodness of fit S are based on F^2^, conventional R-factors R are based on F, with F set to zero for negative F^2^. The threshold expression of F^2^ > 2sigma(F^2^) is used only for calculating R-factors(gt) etc. and is not relevant to the choice of reflections for refinement. R-factors based on F^2^ are statistically about twice as large as those based on F, and R- factors based on ALL data will be even larger.

### Fractional atomic coordinates and isotropic or equivalent isotropic displacement parameters (Å^2^)

**Table:**

	*x*	*y*	*z*	*U*_iso_*/*U*_eq_	
As1	0.351354 (13)	0.98218 (3)	0.284872 (8)	0.01668 (5)	
C1	0.29473 (13)	0.8690 (3)	0.18275 (8)	0.0183 (3)	
C2	0.32226 (14)	0.6566 (3)	0.15347 (9)	0.0226 (3)	
C3	0.27896 (15)	0.5965 (4)	0.07878 (10)	0.0264 (4)	
C4	0.20763 (15)	0.7469 (4)	0.03339 (10)	0.0299 (4)	
C5	0.17979 (17)	0.9578 (4)	0.06212 (10)	0.0313 (4)	
C6	0.22374 (16)	1.0190 (3)	0.13583 (10)	0.0257 (3)	
C7	0.20975 (13)	0.9750 (3)	0.31860 (8)	0.0165 (3)	
C8	0.13587 (13)	0.7891 (3)	0.30118 (9)	0.0192 (3)	
C9	0.03431 (14)	0.7930 (3)	0.32299 (9)	0.0224 (3)	
C10	0.00492 (14)	0.9838 (3)	0.36256 (9)	0.0225 (3)	
C11	0.07749 (14)	1.1692 (3)	0.38019 (9)	0.0235 (3)	
C12	0.17944 (14)	1.1653 (3)	0.35879 (9)	0.0208 (3)	
C13	0.41464 (14)	0.6910 (3)	0.33284 (9)	0.0195 (3)	
C14	0.43828 (15)	0.7124 (3)	0.41818 (9)	0.0218 (3)	
C15	0.48951 (15)	0.4927 (3)	0.45720 (9)	0.0234 (3)	
H2	0.3700 (19)	0.552 (4)	0.1820 (12)	0.026 (6)*	
H3	0.303 (2)	0.458 (4)	0.0593 (13)	0.031 (6)*	
H4	0.1799 (19)	0.704 (4)	−0.0149 (13)	0.040 (6)*	
H5	0.128 (2)	1.066 (5)	0.0299 (15)	0.048 (7)*	
H6	0.2055 (18)	1.172 (4)	0.1569 (12)	0.034 (6)*	
H8	0.1550 (16)	0.654 (4)	0.2729 (11)	0.026 (5)*	
H9	−0.0121 (17)	0.669 (4)	0.3102 (12)	0.033 (6)*	
H10	−0.063 (2)	0.989 (4)	0.3763 (14)	0.033 (7)*	
H11	0.0573 (18)	1.300 (4)	0.4072 (12)	0.036 (6)*	
H12	0.2278 (16)	1.291 (4)	0.3710 (11)	0.024 (5)*	
H13A	0.4768 (18)	0.668 (4)	0.3164 (12)	0.033 (6)*	
H13B	0.364 (2)	0.570 (4)	0.3189 (13)	0.032 (6)*	
H14A	0.370 (2)	0.752 (5)	0.4355 (13)	0.043 (7)*	
H14B	0.4872 (17)	0.844 (4)	0.4344 (11)	0.026 (5)*	
H15A	0.4390 (19)	0.362 (5)	0.4414 (13)	0.037 (6)*	
H15B	0.556 (2)	0.465 (4)	0.4373 (14)	0.032 (6)*	

### Atomic displacement parameters (Å^2^)

**Table:**

	*U*^11^	*U*^22^	*U*^33^	*U*^12^	*U*^13^	*U*^23^
As1	0.01574 (8)	0.01865 (8)	0.01533 (7)	−0.00094 (6)	0.00222 (5)	0.00114 (6)
C1	0.0172 (6)	0.0221 (7)	0.0160 (6)	−0.0017 (6)	0.0044 (5)	0.0026 (5)
C2	0.0218 (7)	0.0269 (8)	0.0194 (7)	0.0002 (7)	0.0050 (6)	0.0007 (6)
C3	0.0285 (9)	0.0296 (9)	0.0225 (8)	−0.0069 (7)	0.0087 (7)	−0.0053 (7)
C4	0.0283 (9)	0.0462 (11)	0.0147 (7)	−0.0115 (8)	0.0028 (6)	−0.0013 (7)
C5	0.0299 (9)	0.0430 (11)	0.0185 (7)	0.0012 (8)	−0.0011 (7)	0.0079 (7)
C6	0.0269 (8)	0.0299 (9)	0.0195 (7)	0.0042 (7)	0.0027 (6)	0.0046 (6)
C7	0.0169 (6)	0.0182 (7)	0.0137 (6)	0.0008 (5)	0.0014 (5)	0.0017 (5)
C8	0.0195 (7)	0.0187 (7)	0.0188 (7)	0.0003 (6)	0.0022 (5)	−0.0016 (5)
C9	0.0204 (7)	0.0243 (8)	0.0217 (7)	−0.0025 (6)	0.0018 (6)	−0.0010 (6)
C10	0.0173 (7)	0.0303 (9)	0.0198 (7)	0.0033 (6)	0.0035 (6)	0.0008 (6)
C11	0.0258 (8)	0.0243 (8)	0.0200 (7)	0.0046 (7)	0.0034 (6)	−0.0030 (6)
C12	0.0219 (7)	0.0195 (7)	0.0200 (7)	−0.0005 (6)	0.0013 (6)	−0.0013 (6)
C13	0.0186 (7)	0.0239 (8)	0.0156 (6)	0.0047 (6)	0.0022 (5)	0.0022 (6)
C14	0.0235 (8)	0.0263 (8)	0.0149 (6)	0.0071 (7)	0.0016 (6)	0.0015 (6)
C15	0.0250 (8)	0.0283 (9)	0.0154 (6)	0.0096 (7)	0.0003 (6)	0.0008 (6)

### Geometric parameters (Å, °)

**Table:**

As1—C1	1.9590 (15)	C8—H8	0.98 (2)
As1—C7	1.9644 (16)	C9—C10	1.391 (2)
As1—C13	1.9688 (16)	C9—H9	0.91 (2)
C1—C2	1.393 (2)	C10—C11	1.386 (3)
C1—C6	1.394 (2)	C10—H10	0.93 (3)
C2—C3	1.400 (2)	C11—C12	1.390 (2)
C2—H2	0.93 (2)	C11—H11	0.95 (2)
C3—C4	1.384 (3)	C12—H12	0.94 (2)
C3—H3	0.94 (2)	C13—C14	1.523 (2)
C4—C5	1.383 (3)	C13—H13A	0.89 (2)
C4—H4	0.91 (2)	C13—H13B	0.93 (3)
C5—C6	1.387 (3)	C14—C15	1.519 (2)
C5—H5	0.99 (3)	C14—H14A	0.98 (2)
C6—H6	0.99 (2)	C14—H14B	0.98 (2)
C7—C8	1.398 (2)	C15—C15^i^	1.525 (3)
C7—C12	1.399 (2)	C15—H15A	0.98 (3)
C8—C9	1.388 (2)	C15—H15B	0.97 (3)
			
C1—As1—C7	96.24 (6)	C10—C9—H9	121.6 (14)
C1—As1—C13	100.26 (7)	C11—C10—C9	119.72 (16)
C7—As1—C13	98.51 (7)	C11—C10—H10	119.9 (13)
C2—C1—C6	118.37 (15)	C9—C10—H10	120.4 (14)
C2—C1—As1	125.33 (12)	C10—C11—C12	120.41 (16)
C6—C1—As1	116.28 (13)	C10—C11—H11	119.8 (14)
C1—C2—C3	120.38 (17)	C12—C11—H11	119.8 (14)
C1—C2—H2	121.8 (14)	C11—C12—C7	120.44 (16)
C3—C2—H2	117.8 (14)	C11—C12—H12	119.7 (12)
C4—C3—C2	120.32 (18)	C7—C12—H12	119.9 (13)
C4—C3—H3	120.8 (14)	C14—C13—As1	111.29 (11)
C2—C3—H3	118.7 (15)	C14—C13—H13A	110.1 (14)
C5—C4—C3	119.60 (16)	As1—C13—H13A	105.9 (15)
C5—C4—H4	121.5 (16)	C14—C13—H13B	108.8 (15)
C3—C4—H4	118.9 (16)	As1—C13—H13B	108.4 (15)
C4—C5—C6	120.16 (18)	H13A—C13—H13B	112 (2)
C4—C5—H5	119.9 (16)	C15—C14—C13	112.84 (14)
C6—C5—H5	119.9 (16)	C15—C14—H14A	110.5 (15)
C5—C6—C1	121.15 (18)	C13—C14—H14A	109.6 (13)
C5—C6—H6	121.0 (13)	C15—C14—H14B	108.6 (12)
C1—C6—H6	117.9 (12)	C13—C14—H14B	110.8 (12)
C8—C7—C12	118.61 (15)	H14A—C14—H14B	104.2 (19)
C8—C7—As1	121.98 (12)	C14—C15—C15^i^	113.73 (18)
C12—C7—As1	119.36 (12)	C14—C15—H15A	107.9 (14)
C9—C8—C7	120.81 (15)	C15^i^—C15—H15A	108.3 (14)
C9—C8—H8	119.0 (12)	C14—C15—H15B	105.7 (14)
C7—C8—H8	120.2 (12)	C15^i^—C15—H15B	113.3 (15)
C8—C9—C10	120.01 (16)	H15A—C15—H15B	107.6 (19)
C8—C9—H9	118.4 (14)		
			
C7—As1—C1—C2	−114.45 (14)	C1—As1—C7—C12	−134.94 (13)
C13—As1—C1—C2	−14.60 (15)	C13—As1—C7—C12	123.67 (13)
C7—As1—C1—C6	67.51 (14)	C12—C7—C8—C9	0.2 (2)
C13—As1—C1—C6	167.36 (13)	As1—C7—C8—C9	−177.24 (12)
C6—C1—C2—C3	−0.2 (2)	C7—C8—C9—C10	0.1 (2)
As1—C1—C2—C3	−178.21 (13)	C8—C9—C10—C11	−0.1 (2)
C1—C2—C3—C4	−0.5 (3)	C9—C10—C11—C12	−0.2 (3)
C2—C3—C4—C5	0.4 (3)	C10—C11—C12—C7	0.6 (2)
C3—C4—C5—C6	0.5 (3)	C8—C7—C12—C11	−0.6 (2)
C4—C5—C6—C1	−1.2 (3)	As1—C7—C12—C11	176.96 (12)
C2—C1—C6—C5	1.0 (3)	C1—As1—C13—C14	−165.02 (12)
As1—C1—C6—C5	179.24 (15)	C7—As1—C13—C14	−67.05 (13)
C1—As1—C7—C8	42.50 (13)	As1—C13—C14—C15	−178.78 (13)
C13—As1—C7—C8	−58.90 (13)	C13—C14—C15—C15^i^	−178.14 (19)

Symmetry codes: (i) −*x*+1, −*y*+1, −*z*+1.

### Hydrogen-bond geometry (Å, °)

**Table:**

*D*—H···*A*	*D*—H	H···*A*	*D*···*A*	*D*—H···*A*
C15—H15B···Cg1^ii^	0.97 (3)	2.81 (3)	3.776 (2)	169.9 (18)
C4—H4···Cg2^iii^	0.91 (2)	2.80 (2)	3.708 (2)	173.2 (19)
C9—H9···Cg2^iv^	0.91 (2)	2.97 (2)	3.617 (2)	129.5 (16)

Symmetry codes: (ii) −*x*+1, *y*−1/2, −*z*+1/2; (iii) *x*, −*y*+1/2, *z*−3/2; (iv) −*x*, *y*−1/2, −*z*+1/2.
